# Practices, Potential, and Perspectives for Detecting Predisease Using Raman Spectroscopy

**DOI:** 10.3390/ijms241512170

**Published:** 2023-07-29

**Authors:** Yusuke Oshima, Takayuki Haruki, Keiichi Koizumi, Shota Yonezawa, Akinori Taketani, Makoto Kadowaki, Shigeru Saito

**Affiliations:** 1 Faculty of Engineering, University of Toyama, Toyama 930-8555, Japan; 2 Research Center for Pre-Disease Science, University of Toyama, Toyama 930-8555, Japan; 3 Faculty of Medicine, Oita University, Yufu 879-5593, Japan; 4 Faculty of Sustainable Design, University of Toyama, Toyama 930-8555, Japan; 5 Division of Presymptomatic Disease, Institute of Natural Medicine, University of Toyama, Toyama 930-8555, Japan

**Keywords:** Raman spectroscopy, molecular fingerprint, predisease, clinical application, state transition, dynamical network biomarker, DNB

## Abstract

Raman spectroscopy shows great potential for practical clinical applications. By analyzing the structure and composition of molecules through real-time, non-destructive measurements of the scattered light from living cells and tissues, it offers valuable insights. The Raman spectral data directly link to the molecular composition of the cells and tissues and provides a “molecular fingerprint” for various disease states. This review focuses on the practical and clinical applications of Raman spectroscopy, especially in the early detection of human diseases. Identifying predisease, which marks the transition from a healthy to a disease state, is crucial for effective interventions to prevent disease onset. Raman spectroscopy can reveal biological processes occurring during the transition states and may eventually detect the molecular dynamics in predisease conditions.

## 1. Introduction

In 1928, Chandrasekhara Venkata Raman made a groundbreaking discovery known as the Raman effect in India [1,2]. The emergence of lasers solidified the analytical potential of Raman spectroscopy, transforming it into an invaluable tool for visualizing the structure and composition of molecules in cells and tissues. This label-free technique leverages the wavelength shift of scattered light (Raman scattered light), which originates from the interaction between the intrinsic vibration of molecules and the vibration of the electric field of incident light. 

Since then, Raman spectroscopy has been applied in various fields to analyze the chemical and molecular structures of organic and inorganic materials [3]. It has a wide range of applications, from batteries, displays, and electronic devices to food, pharmaceuticals, and biotechnology. Raman spectroscopy is useful for component analyses of carbon, semiconductors, polymers, pharmaceutical materials, and for crystallinity/stress evaluations. This technique has been applied in basic medical research since the 1980s [4,5,6]. In the 2000s, numerous papers reported its usefulness as a diagnostic technique for cancers, arteriosclerosis, Alzheimer’s disease, etc. [7,8,9,10,11,12,13,14,15,16,17]. For biological specimens such as cells, tissues, and organs, Raman spectroscopy is typically sensitive to concentrations of biomolecules such as lipids, proteins, carbohydrates, and nucleic acids. However, the scattering light is extremely weak, limiting accurate measurements and practical applications. Recent advances in laser light sources and optical measurement technologies have made clinical applications, such as tissue biopsy, cytology, and intraoperative pathology diagnosis feasible [18,19,20].

Raman spectroscopy can accurately and quickly identify the resection margin of a lesion for intraoperative pathology diagnosis, reducing patient burden and preventing postoperative complications. In addition, introducing the Raman technique to endoscopy, laparoscopy, and arthroscopy should realize the early diagnosis and early intervention in treatment of disease. However, Raman spectroscopy only provides information from molecular vibrations, making it extremely difficult to understand the biological significance of signals from multi-component samples such as cells and tissues. Raman spectroscopy, on the other hand, is a nondestructive analytical tool, and can capture state transitions in biological activities, providing a methodology to measure biological fluctuations from molecular vibrations that was previously unavailable [21]. 

This review overviews the technical advances in biomedical applications of Raman spectroscopy and the latest research results in cells and tissue diagnostics, including biopsies for human disease. Specifically, the advances in measuring biological fluctuations are discussed from the aspect of molecular vibrations. Moreover, the potential to detect predisease and realize the early intervention and prevention of disease by mathematically understanding the state before disease onset may be a solution to the challenge of extending a healthy life expectancy in an aged society.

## 2. Principle of Raman Scattering and Instrumentation

**Figure 1:**
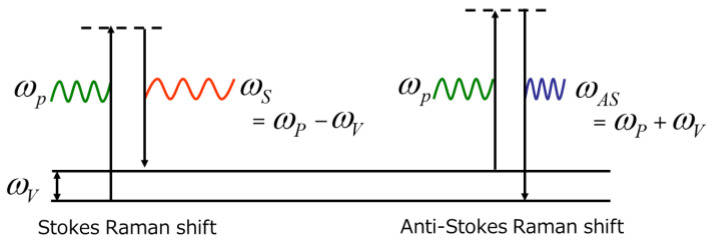
Jablonski diagram showing the energy transition of the Raman scattering process [3,13]. ω_P_ indicates the frequency of the pump light; ω_V_ corresponds to the vibrational frequency of molecules in the electric field; and ω_S_ and ω_AS_ are the frequency of Stokes and anti-Stokes Raman scattering, respectively.

Raman spectroscopy is a technique for analyzing the structure and composition of molecules from this Raman spectrum. Another vibrational spectroscopic method is IR (infrared) spectroscopy. In an IR spectroscopy measurement, the photon energy of absorption directly corresponds to the molecular vibration frequency. In contrast, Raman spectroscopy detects the difference in energy between the pump light and vibrational energy of the molecules during the excitation to a higher vibrational level. This difference is shown in a Jablonski diagram (Figure 1). An advantage of Raman spectroscopy is that the pump light should be monochromatic, but a wide wavelength range from UV to NIR can be used independent of the sample states (solid, liquid, and gas), media-containing sample (in water, air, and vacuum) and situation (transmission, back and forward scattering, etc.).

**Figure 2:**
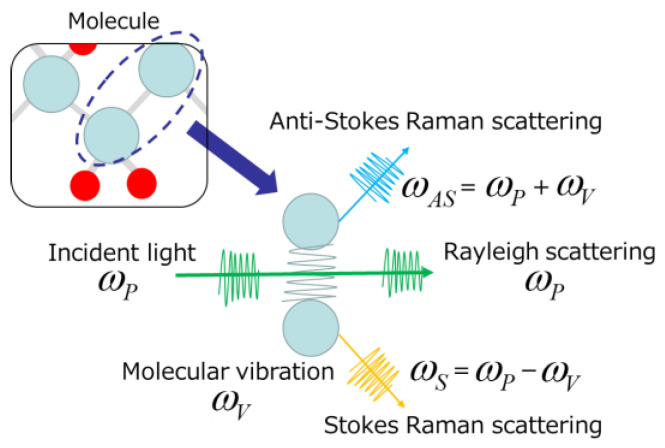
Schematic depicting the principle of Raman scattering [3,13].

Irradiating a material with monochromatic light (e.g., a fixed-wavelength laser) simultaneously scatters light with the same wavelength as the incident light (Rayleigh scattering light) and light with a slightly different wavelength [1,3,13]. The light scattered with a frequency shift relative to the incident light is called Raman scattering (Figure 2). Stokes Raman scattering occurs when the scattering light shifts to the longer-wavelength side (smaller frequency; ω_P_ −ω_V_), whereas that shifting to the shorter wavelength side is called anti-Stokes Raman scattering (larger frequency; ω_P_ + ω_V_) (Figure 1 and Figure 2). Since Stokes Raman scattering is usually stronger, the term “Raman scattering” often refers to Stokes Raman scattering. Raman scattering reflects the vibrational energy in the electric field of molecules and gives a scattering spectrum (Raman spectrum) specific to the molecules. 

**Figure 3:**
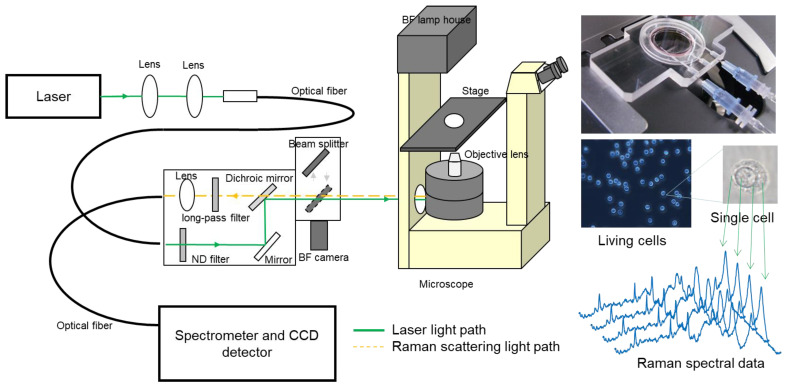
Schematic illustration of typical Raman microscopy system for living cells and tissue sections [22,24].

Several methods can be used to measure Raman spectra. However, an excitation laser is typically used to measure solid samples such as materials or biological specimens. After passing the backscattered or forward light through a notch filter or long-pass filter to cut the Rayleigh scattered light, a multichannel spectrometer gives the spectrum of the scattered light (Figure 3). Although infrared absorption spectroscopy is a common vibrational spectroscopic method to identify chemical substances, it is not well suited for biological measurements due to the extremely high absorption of water in infrared light. In contrast, Raman spectroscopy provides information that is almost equivalent to the IR absorption spectrum, since it can employ visible to near-infrared light, which is unaffected by water absorption. Hence, Raman spectroscopy is well-suited for biological tissue measurements, and especially Raman microscopy is a powerful tool for analyzing a living cell in situ with custom-designed instrumentation and devices [22,23,24].

Another advantage is that direct analysis from the Raman spectra is feasible since target molecules do not have to be labeled beforehand. Consequently, it should be applicable to clinical applications such as rapid and in situ diagnostic techniques for cancer, which have been difficult to achieve using highly invasive conventional pathological diagnosis and biopsies. Unfortunately, the signal intensity of Raman scattering from biological samples is extremely small. Advanced measurement techniques are necessary because compared to the absorption cross section for photon, the Raman scattering cross section is extremely small, typically from 10^−30^ to 10^−25^ cm^2^ [25]. Recently, the increased sensitivity of CCD sensors and the miniaturization and diversification of excitation laser sources have reduced these technical hurdles [3]. Today, Raman spectroscopy is more accessible to basic medical researchers and clinical doctors because it is becoming more versatile and economical. Since its discovery almost a century ago, Raman spectroscopic analysis technology has continued to progress.

## 3. Technical Breakthroughs toward Biomedical Applications

In the past three decades, Raman spectroscopy has been widely utilized in biomedical research and clinical applications. The greatest advantage of Raman spectroscopy in clinical use is it is minimally invasive while providing objective information based on the molecular composition in cells, tissues, and organs. In 1990, Puppels et al. published an original article about measuring the Raman spectra of single living cells and analyzing the molecular distribution in a chromosome [26]. They developed confocal Raman microspectroscopy, enabling high-resolution spectral data to be acquired from single cells. They successfully interpreted the origin of the Raman peaks (now the so-called peak assignment) observed in human granulocytes [27]. At the same time, histopathological applications were also reported. Baraga et al. investigated atherosclerotic lesions of the human artery using FT-Raman spectroscopy [28].

In 2000, Shim et al. demonstrated the first in vivo Raman spectroscopic measurement of human gastrointestinal tissues during a routine clinical endoscopy when they reported their fiber-optic Raman spectroscopic system as a rapid communication [29]. Since the 2000s, physical chemists, analytical chemists, medical engineers, and physicians have conducted in vitro, ex vivo, and in vivo research using Raman spectroscopic techniques [8,9,10,16,17,30,31,32]. 

The modality of Raman spectroscopy has also diversified since 2000. Hamada et al. developed a line-scanning Raman microscopy system, which yields highly spatial- and time-resolved Raman spectral data from living cells [33]. Today, Raman spectroscopy is known not only as a spectroscopic method but also as a bioimaging technique and confocal fluorescence microscopy. Nonlinear Raman scattering, coherent anti-Stokes Raman scattering (CARS), and stimulated Raman scattering (SRS) methods can stimulate the excitation of coherent motions of vibrational oscillators, offering vibrational imaging with subcellular spatial resolution and an image acquisition speed of more than four orders of magnitude higher than that of spontaneous Raman microscopy [34,35,36,37,38,39,40].

In 2010, Saar et al. improved in vivo SRS imaging by substantially enhancing the collection of the backscattered signal and increasing the imaging speed by three orders of magnitude, which is comparable to the video rate [34]. At the same time, Ozeki et al. reported an SRS microscopy system for video-rate live cell imaging [35]. They also demonstrated its potential for use in histopathological applications by assessing the tissue section of an acetaminophen-overdosed mouse liver and capturing the pathological changes, including centrilobular necrosis [36]. CARS techniques can provide cellular and tissue spectral images in live cell culture and disease models for multiple sclerosis [38,39,40,41]. The significant merits of nonlinear Raman spectroscopic techniques are high-speed measurements and the acquired high-contrast images. Other applications include intraoperative diagnostics [42], imaging flow cytometry [43], and cell sorting [44]. 

A Raman image-based approach emphasizes the limited number of Raman bands assigned to moieties in lipids and proteins. However, they sacrifice the spectral information of other molecules in cells and tissues. On the other hand, spontaneous Raman techniques give a lot of molecular information in the fingerprint region of biological samples, but the signal collection efficiency is relatively poor. As a diagnostic tool for the early detection of disease, spontaneous Raman techniques have the potential for practical clinical applications [45,46,47,48,49,50,51,52,53,54,55,56,57,58,59,60,61,62,63,64,65,66]. Furthermore, surface-enhanced Raman scattering (SERS), spatially offset Raman spectroscopy (SORS), and incorporating machine learning are complementary technologies that increase the detection sensitivity in liquid biopsies [63,64,65] and deep layers of tissues and organs [58,66]. They also increase accuracy in discriminant analysis [67,68,69,70,71,72].

## 4. Molecular Fingerprints Possibly Associated with Diseases on the Raman Spectrum Obtained from Cells and Tissues

As mentioned above, Raman spectroscopy provides information regarding molecular vibrations, known as “molecular fingerprints.” The fingerprint region includes the most characteristic groups of vibrations for each molecule. It typically comprises 900–2200 cm^−1^ IR region and excludes stretching C-H vibrations in the region 2900–3100 cm^−1^ which are not very informative. Recent discrete Fourier transform (DFT) calculations allow an increase in the important and useful normal mode frequency up to the THz region, which is available for Raman scattering (but not used in FT-IR spectra). Thus, the concept of fingerprint vibrations used originally in IR spectroscopy nowadays is changed concerning the Raman application. The role of low-frequency vibrations is important for docking studies, for intermolecular interactions between drugs and receptors. Together with DFT and molecular mechanic calculations, such low-frequency Raman spectroscopy provides a unique ability in molecular medicine [73,74,75,76]. In addition, analyzing those fingerprints may offer valuable insights into the early detection of diseases by tracking the structural and compositional changes in molecules that occur in physiological processes during disease development. To date, there is a great clinical interest in developing a rapid and non-invasive methodology that enables the real-time monitoring of the molecular dynamics occurring in living cells and tissues during disease onset, overcoming the limitations of conventional biochemical techniques. Here, we are focusing on the typical Raman peaks of human cells and tissues involved in molecular fingerprints associated with diseases by the past literature survey. 

Generally, in the case of cellular analysis on Raman spectroscopy, strong Raman peaks at 1754 cm^−1^ (C=O), 1656 cm^−1^ (C=C), 1440 cm^−1^ (CH_2_ bend), and 1300 cm^−1^ (CH_2_ twist) can be observed as the fingerprint of lipid contents. The characteristics of protein contents can also be understood from the Raman peaks at 1656 cm^−1^ (amide I), 1615 cm^−1^ at (tyrosine and tryptophan), 1450 cm^−1^ (CH_2_ bend), 1100–1375 cm^−1^ (amide III), and 1004 cm^−1^ (phenylalanine). In addition, the contents of nucleic acid contribute around 785 cm^−1^ and around 1094 cm^−1^ due to PO_2_ backbone vibrations [3,26,33,45]. Since DNA (RNA) has four nucleobases, prominent Raman bands at 730 cm^−1^, 785 cm^−1^, 1340 cm^−1^, 1490 cm^−1^ and 1580 cm^−1^ are attributed to the base composition in nucleotides (e.g., ring breathing modes in the DNA bases) [77,78,79,80]. Cytosine and thymine (uracil) are pyrimidine derivatives, adenine and guanine are purine derivatives, consisting of a fused pyrimidine-imidazole ring system with conjugated double bonds. Those conjugated moieties including aromatic amino acid residues contribute characteristics of molecular fingerprint measuring cellular components as well.

Movasaghi et al. reported in 2007 that Raman spectral interpretation and detailed peak assignments were collected to provide a database of molecular fingerprints for defining the chemical structure of the biological tissues, introducing most of the important peaks present in natural tissues [81]. Since then, the Raman spectral fingerprints were utilized for cancer cell detection and discrimination.

Harvey et al. demonstrated that the spectral discrimination of live prostate cancer cells (PC-3) and bladder cancer cells (MGH-U1) was performed by using Raman optical tweezers. From these Raman spectral fingerprints and the assignments for contributed molecular vibrations, proteins and nucleic acids could be more abundant in MGH-U1 than PC-3 cells, while lipids and carbohydrates were more abundant in PC-3 cells. Nucleic acids and proteins were mainly found in the cell nucleus, while lipids are largely distributed within the cytoplasm and cell membrane. Therefore, differences in these biochemical amounts between PC-3 and MGH-U1 may be a consequence of differences in the nucleus-to-cytoplasm ratio between these cells. The Raman spectral fingerprint of each cell could be linked to cell size, as the proportion of the nucleus and cytoplasm probed is likely to vary with cell size [82].

If the energy of the excitation laser happens to coincide with an electronic transition within the molecule, Raman scattering can be greatly enhanced, this phenomenon is known as resonance Raman scattering. Even excitation close to the electronic transition of a molecule can yield “pre-resonance.” Several molecules which have conjugated double bonds present quite strong Raman peaks in living cells due to resonant Raman scattering. The Raman spectral features of erythrocytes are unique and easily distinguished from other cells. By using a 632.8 nm excitation laser, oxygenated, deoxygenated, and metHb-erythrocytes can be characterized due to their own molecular fingerprints on Raman spectral features [83]. Cytochrome c also shows relatively strong and sharp resonant Raman peaks by measuring with a 532 nm excitation. It is useful to explore the cellular distribution of mitochondria on Raman microscopy [84,85,86]. Cytochrome c is a key molecule that maintains respiratory function and cell apoptosis. Oshima et al. reported discrimination analysis of the different histological types of lung cancer cell lines using molecular fingerprints of each cell type, and they found that the relative peak intensities of cytochrome c between cancer cells and normal cells were significantly different [45]. Okada et al. performed the label-free observation of molecular dynamics in apoptotic cells using a Raman microscope and successfully captured the dynamic changes in the cytochrome c distribution at the Raman band of 750 cm^−1^, which was assigned to pyrrole breathing mode ν_15_ in cytochrome c, after adding an apoptosis inducer to the cells [85]. Recently, Abramczyk et al. reported Raman spectroscopy and imaging to monitor changes in the redox state of the mitochondrial cytochromes in ex vivo surgically resected specimens of human breast tissues, and in vitro human breast cells of normal cells. They found that the global concentration of cytochrome c in the breast tissue (reflected by the Raman intensity of the bands at 1584 cm^−1^ and 750 cm^−1^) increases with cancer aggressiveness [86]. 

Considering the expansion of the measurement target from the single cell level to the tissue level, the molecular species which are involved in molecular fingerprint features obtained from Raman spectroscopy become more abundant due to the existence of extracellular matrix (ECM). Haka et al. reported that they employed Raman spectroscopy to diagnose benign and malignant lesions in human breast tissue based on its chemical composition, including the epithelial cell cytoplasm, cell nucleus, fat, β-carotene, collagen, calcium hydroxyapatite, calcium oxalate dihydrate, cholesterol-like lipid deposits, and water. This approach was based on the assumptions that the Raman spectrum of a mixture is a linear combination of those Raman spectra assigned to the molecular components, and yields a sensitivity of 94% (29/31), a specificity of 96% (91/95), and an overall accuracy of 86% (108/126) for detecting infiltrating carcinoma [87].

Raman spectroscopy also has the potential to further our understanding of cardiovascular calcification. You et al. performed Raman spectroscopy imaging to examine the molecular composition and spatial distribution of the mineral and organic content in human aortic tissue cross-sections. The representative molecular fingerprint involved in specific components of the aortic tissue, including elastin, collagen, lipid (cholesterol), β-carotene, apatite, and whitlockite, were identified for characterizing atherosclerosis [88]. The molecular fingerprint in vascular tissues may offer possible mechanisms and the early detection of cardiovascular diseases such as atherosclerosis and aortic stenosis (AS) [16,17,88]. 

The molecular composition in the bone and cartilage matrix is also a suitable target for tissue Raman measurement due to the abundance of ECM. The excitation laser of 785 nm is often to reduce the autofluorescence background, but 532 nm is also available, and the result could be consistent. Raman spectroscopic fingerprints in bone tissue could contribute to predicting fracture risk in osteoporosis [89,90]. Several Raman peaks originating from the inorganic and organic components of the bone matrix could be identified. The bone matrix mainly consists of hydroxyapatite and type I collagen. The spectral feature is dominated by apatite phosphate groups (O-P-O) symmetric stretching at 961 cm^−1^, while the peak at 1070 cm^−1^ is evidence of the presence of carbonates. The main protein vibrational modes appear in the ranges 1150–1350 cm^−1^ (amide III) and 1630–1690 cm^−1^ (amide I) and correspond to different vibrations of the peptide bonds, which are sensitive to the protein secondary structure. Some bands typical of single amino acids can be also recognized (phenylalanine 1005 cm^−1^ and proline 855 cm^−1^). The Raman spectroscopic fingerprint of bone, which can be characterized by its mineral/matrix ratio (1005 cm^−1^/961 cm^−1^), carbonate/phosphate ratio (1070 cm^−1^/961 cm^−1^), collagen crosslink maturity (e.g., 1660/1690 in amide I), and crystallinity (e.g., peak width at 961 cm^−1^), can become a valuable surrogate marker in fracture risk assessment and the evaluation of therapeutics in osteoporosis [89,90,91]. Some reports suggest that the Raman spectroscopic fingerprint of the cartilage matrix makes it possible for early detection and prognostic prediction in osteoarthritis [24,92,93]. The main components of articular cartilage are glycosaminoglycans (GAGs) and type II collagen, and changes in them can be used as indicators for the early diagnosis of OA. Kumar et al. showed the contents of amide I (1612–1696 cm^−1^) and protein decrease with the increasing severity of OA [92]. Asaoka et al. elucidated a negative correlation between clinical OA grading and the peak intensities at 1042 cm^−1^ (C-O-C) and 1061 cm^−1^ (O-SO_3_^−^) which could be assigned to GAGs contents in the cartilage matrix [24].

Raman fingerprinting often provides reliable information directly associated with the diagnostic and prognostic markers for disease (e.g., cancers, atherosclerosis, osteoporosis, and OA). However, to fully exploit the fingerprint for clinical use, further analytical strategies including hardware technologies and methodologies in machine learning should be introduced.

## 5. Recent Advances and Limitations in Clinical Applications of Raman Spectroscopy

As the technology has matured in the past few decades, the annual number of research papers on Raman spectroscopic and imaging applications for clinical diagnostics has drastically increased. The literature involving Raman spectroscopy and human diseases is too numerous to list so representative articles are selectively cited here (Table 1). For the early detection and prediction of human disease, applications range from the discriminant analysis of cancer cells [72,94], tissues [51,53,62,69,71,95,96,97], and serum sample [67,98] to diagnostic procedures via endoscopy [95]. Cheng et al. demonstrated that four leukocyte types (granulocytes, monocytes, B cells, and T cells) from healthy people were characterized as a reference of normal hematopoiesis and were distinguished from each other by generating an orthogonal partial least squares discriminant analysis (OPLS-DA) model for the further analysis of leukemic granulocytes [72]. They found that a combination of the Raman peaks at 1003, 1341, and 1579 cm^−1^ Raman peaks could discriminate myeloblasts and abnormal promyelocytes from normal granulocytes and verified with 92.59% accuracy. These excellent diagnostic results have been achieved by utilizing multivariate analyses and decision algorithms. 

In general, Raman spectral data obtained from biological samples are very complicated. As discussed above, the molecular fingerprinting based on Raman spectroscopic measurement may contribute to early detection and prognostic prediction in some cases but identifying or discriminating the Raman spectral fingerprint in each state (e.g., healthy, predisease, disease) is still challenging task in the practical situation. Multivariate analytical methods, principal component analysis (PCA), and partial least square (PLS) regression analysis have been used for a long time. These exploratory analyses provide objective interpretations of the Raman spectral changes in disease. Since the gold standard for disease diagnosis is a histological assessment of suspicious cells, tissues, and blood samples obtained from patients, the disease states determined by Raman spectroscopy must be confirmed by routine histopathology. Although such a limitation exists, recent advances in machine learning methods have linked characteristics in Raman spectral data to known pathological states more effectively and rapidly. High-throughput Raman spectroscopy combined with fine-tuned machine learning is potentially useful for the early detection and prognostic diagnosis in human disease [96,97].

## 6. From “Discriminant Analysis” to “Transition-State Analysis”

Identifying biomarkers for early detection and prognostic prediction in diseases is a challenge in current Raman spectroscopic approaches only utilizing exploratory analysis and sophisticated decision algorithms because today’s medical science does not have a method to define the predisease state [99]. To address the issue, Aihara et al. suggested a clear and quantitative definition of predisease states from a mathematical viewpoint as critical states just before bifurcation points from healthy to disease states. They proposed a theoretical methodology to detect early warning signals peculiar to the predisease states with dynamical network biomarkers (DNB) [100,101]. 

**Figure 4:**
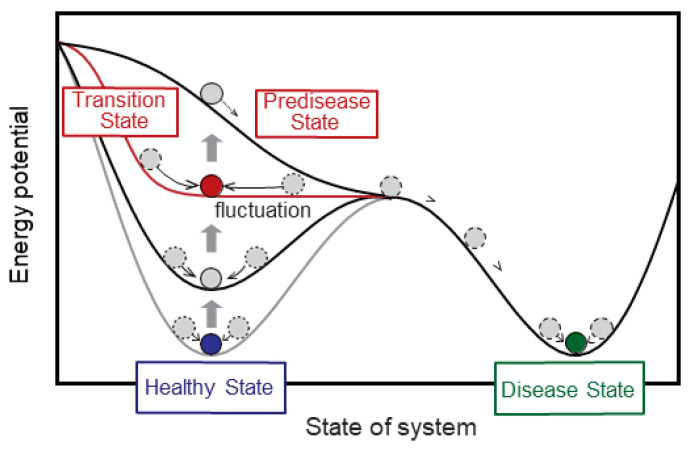
Definition of the predisease state based on DNB theory [21,99].

Figure 4 depicts a conceptual diagram of healthy, predisease, and disease states by a hypothetical potential function. The state of the body or biological system slowly changes from a healthy state to the predisease state, but then it suddenly moves to a disease state after the transition or bifurcation. The red curve shows the hypothetical potential function with the transition state just before the predisease state. 

**Figure 5:**
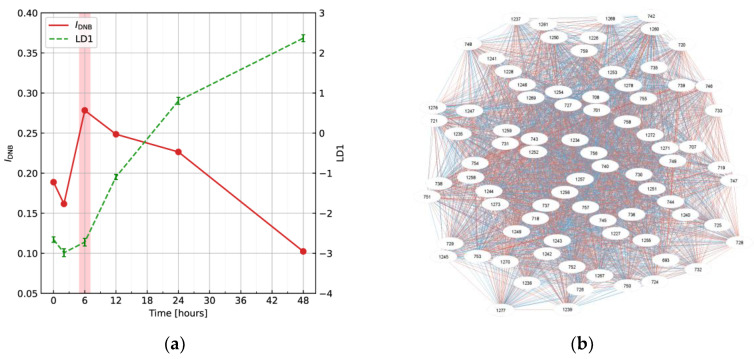
Numerical results by the application of the DNB theory: (**a**) the time evolution of ordinal and dynamical network biomarkers; and (**b**) the network of DNB elements. These figures were retrieved from Haruki et al. [21].

Figure 5 shows the numerical results by the application of the DNB theory to Raman spectra in the mouse T cell activation process [21]. As seen in Figure 5a, normally, the ordinal biomarker (green dashed line) gradually increases, resulting in the difference between two states: naïve and activated states, corresponding generally to health and disease states. The marker corresponds to the score of linear discriminant analysis to distinguish these states. On the other hand, the DNB score (red solid line) calculated from the fluctuations in and correlations between elements (variables) in a complex network reveals the onset of the transition state as a peak at a specified time point. Therefore, the DNB theory successfully contributes to detecting early warning signals, which are not found in conventional biomarkers.

The DNB score is defined by the product of averaged standard deviation and correlation strength of variables in DNB candidate groups [99,100,101]. First, evaluating the variance on each variable completely separates largely fluctuating and non-fluctuating variables. Then, the correlation strength *r_i,j_* between variables *x_i_* and *x_j_* represents the edge between nodes. It is sufficient to calculate the correlation coefficients between variables with large fluctuations to extract DNB candidate groups. Here, correlations are shown as edges and the red and blue edges show positive and negative correlations, respectively. Therefore, the groups including largely fluctuating and highly correlated variables are extracted as DNB candidate groups (See Figure 5b) using hierarchical clustering. Here, the clusters to be extracted are the largest clusters in size, or the second and subsequent clusters larger than half of the size. Finally, DNB candidate groups become DNB when the DNB score shows the peak at a specified time point. The time point corresponds to the onset of the transition state. In conjunction, variables included in the DNB candidate groups are selected as DNB elements. 

Koizumi et al. first reported that DNBs used to predict metabolic syndrome were successfully identified from the dataset of time-course gene expression profiles in an animal model [102]. To date, studies on DNB analysis for gene expression levels have been conducted, including this report [99,102,103]. Predictive signs (very early signals) have been detected in a variety of diseases. However, these gene expression data are obtained by destructive testing involving the sacrifice of animals such as mice, which poses a major problem when considering its application to humans. Raman spectroscopy is a better-suited methodology to detect a transition state associated with the predisease state for potential clinical applications. Haruki et al. applied the DNB theory to Raman spectra of T cell activation [21]. In this case, the initial and final states corresponded to naïve and fully activated T cells, respectively. This article suggested that a combination of DNB theory and Raman spectroscopy provides additional information, which cannot be found in current multivariate analyses, to estimate the transition state. This is the first model case and trial to detect the transition state and identify DNB Raman shifts exhibiting abnormal fluctuations at the transition state.

This approach is not restricted to biomarkers such as gene expression profiles and Raman spectra. It is also important to mathematically capture the signs (early warning signals) of state transitions such as ecosystem changes and stock fluctuations. Once these signs are known, countermeasures can be implemented. In the case of disease, lifestyle modifications or medical interventions can be made before the disease becomes serious, thereby extending a healthy life expectancy.

## 7. Conclusions and Future Directions

This review should help realize applications using conventional analysis techniques and devising an innovative measurement technology platform to detect predisease. In the future, the early detection and prevention of diseases and an intervention to prevent the appearance of disease may be possible by elucidating the transition state using Raman spectroscopy. Furthermore, Raman microscopy and DNB theory may detect the unaffected state of clinical specimens from their Raman scattering spectra.

## Figures and Tables

**Table 1:** The representative literature of Raman spectroscopic studies for human diseases.

Target Disease	Sample Type (Modality)	Analytical Method	Reference
colon cancer	in vivo (endoscopy)	PCA, neural network	Shim et al., 2000 [29]
	ex vivo (fiber probe)	PCA-LDA	Molchovsky et al., 2003 [30]
	in vivo (endoscopy)	PLS-DA	Belgholt et al., 2010 [46]
	in vivo (endoscopy)	PCA, DT, AdaBoost	Fousková et al., 2023 [95]
lung cancer	ex vivo (microscopy)	Histogram	Yamazaki et al., 2003 [9]
	in vivo (fiber probe)	intensity ratio	Huang et al., 2003 [10]
	in vitro (microscopy)	PCA	Oshima et al., 2010 [45]
	ex vivo (microscopy)	CNN	Qi et al., 2022 [69]
breast cancer	ex vivo (microscopy)	PCA	Haka et al., 2002 [8]
	in vivo (fiber probe)	x^2^ analysis	Haka et al., 2006 [31]
	ex vivo (microscopy)	CNN	Ma et al., 2021 [51]
	ex vivo (fiber probe)	SVM, Lasso	David et al., 2023 [97]
esophageal cancer	ex vivo (fiber probe)	PLSR, SOMs, LDA	Ishigaki et al., 2016 [49]
bladder cancer	in vivo (fiber probe)	PC-GDA	Lui et al., 2012 [56]
skin cancer	ex vivo (microscopy)	ResNet50	Chen et al., 2022 [71]
gastric cancer	in vivo (endoscopy)	PCA, PLS-DA	Duraipandian et al., 2012 [47]
brain tumor	ex vivo (microscopy)	PCA, PLS, LDA	Aguiar et al., 2022 [55]
liver cancer	ex vivo (microscopy)	CNN	Huang et al., 2023 [62]
cervical cancer	ex vivo (microscopy)	CNN	Kang et al., 2023 [96]
thyroid cancer	blood serum (microscopy)	SMOTE	Song et al., 2021 [67]
leukemia	blood smear (microscopy)	PLS-DA, SVM	Féré et al., 2019 [50]
	blood serum (microscopy)	PLS-DA	Lima et al., 2022 [98]
	bone marrow cells (microscopy)	OPLS-DA	Cheng et al., 2022 [72]
prostate cancer bone metastasis	in vitro (microscopy)	PCA	Kar et al., 2022 [94]
atherosclerosis	in vitro (FT-Raman)	PCA	Nogueira et al., 2005 [16]
	in vitro (fiber probe)	MCR	Sćepanović et al., 2006 [17]
	ex vivo (microscopy)	VCA image unmixing	You et al., 2017 [88]
dry eye	meibum lipid (microscopy)	PCA	Oshima et al., 2009 [32]
osteoarthritis	ex vivo (microscopy)	PCA	Kumar et al., 2015 [92]
	ex vivo (microscopy)	PCA, HCA	Asaoka et al., 2022 [24]
	in vivo (needle arthroscopy)	PLS-DA	Kroupa et al., 2021 [93]
Hirschsprung disease	ex vivo (microscopy)	PCA	Ogawa et al., 2021 [54]

## Data Availability

Not applicable.

## References

[1] Raman C.V., Krishnan K.S. (1928). A New Type of Secondary Radiation. Nature.

[2] Landsberg G.S., Mandelstam L.I. (1928). Über die Lichtzerstreuung in Kristallen. Z. Für Phys..

[3] Shipp D.W., Sinjab F., Notingher I. (2017). Raman spectroscopy: Techniques and applications in the life sciences. Adv. Opt. Photonics.

[4] Ozaki Y., Mizuno A., Itoh K., Iriyama K. (1987). Inter- and intramolecular disulfide bond formation and related structural changes in the lens proteins. A Raman spectroscopic study in vivo of lens aging. J. Biol. Chem..

[5] Bot A.C., Huizinga A., de Mul F.F., Vrensen G.F., Greve J. (1989). Raman microspectroscopy of fixed rabbit and human lenses and lens slices: New potentialities. Exp. Eye Res..

[6] Clarke R.H., Isner J.M., Gauthier T., Nakagawa K., Cerio F., Hanlon E., Gaffney E., Rouse E., DeJesus S. (1988). Spectroscopic characterization of cardiovascular tissue. Lasers Surg. Med..

[7] Bakker Schut T.C., Witjes M.J., Sterenborg H.J., Speelman O.C., Roodenburg J.L., Marple E.T., Bruining H.A., Puppels G.J. (2000). In vivo detection of dysplastic tissue by Raman spectroscopy. Anal. Chem..

[8] Haka A.S., Shafer-Peltier K.E., Fitzmaurice M., Crowe J., Dasari R.R., Feld M.S. (2002). Identifying microcalcifications in benign and malignant breast lesions by probing differences in their chemical composition using Raman spectroscopy. Cancer Res..

[9] Yamazaki H., Kaminaka S., Kohda E., Mukai M., Hamaguchi H.O. (2003). The diagnosis of lung cancer using 1064-nm excited near-infrared multichannel Raman spectroscopy. Radiat. Med..

[10] Huang Z., McWilliams A., Lui H., McLean D.I., Lam S., Zeng H. (2003). Near-infrared Raman spectroscopy for optical diagnosis of lung cancer. Int. J. Cancer.

[11] Koljenović S., Bakker Schut T.C., van Meerbeeck J.P., Maat A.P., Burgers S.A., Zondervan P.E., Kros J.M., Puppels G.J. (2004). Raman microspectroscopic mapping studies of human bronchial tissue. J. Biomed. Opt..

[12] Chou I.H., Benford M., Beier H.T., Coté G.L., Wang M., Jing N., Kameoka J., Good T.A. (2008). Nanofluidic biosensing for beta-amyloid detection using surface enhanced Raman spectroscopy. Nano Lett..

[13] Hanlon E.B., Manoharan R., Koo T.W., Shafer K.E., Motz J.T., Fitzmaurice M., Kramer J.R., Itzkan I., Dasari R.R., Feld M.S. (2000). Prospects for in vivo Raman spectroscopy. Phys. Med. Biol..

[14] Juszczak L.J. (2004). Comparative vibrational spectroscopy of intracellular tau and extracellular collagen I reveals parallels of gelation and fibrillar structure. J. Biol. Chem..

[15] Motz J.T., Gandhi S.J., Scepanovic O.R., Haka A.S., Kramer J.R., Dasari R.R., Feld M.S. (2005). Real-time Raman system for in vivo disease diagnosis. J. Biomed. Opt..

[16] Nogueira G.V., Silveira L., Martin A.A., Zângaro R.A., Pacheco M.T., Chavantes M.C., Pasqualucci C.A. (2005). Raman spectroscopy study of atherosclerosis in human carotid artery. J. Biomed. Opt..

[17] Sćepanović O.R., Fitzmaurice M., Gardecki J.A., Angheloiu G.O., Awasthi S., Motz J.T., Kramer J.R., Dasari R.R., Feld M.S. (2006). Detection of morphological markers of vulnerable atherosclerotic plaque using multimodal spectroscopy. J. Biomed. Opt..

[18] Egawa M. (2021). Raman microscopy for skin evaluation. Analyst.

[19] Noothalapati H., Iwasaki K., Yamamoto T. (2021). Non-invasive diagnosis of colorectal cancer by Raman spectroscopy: Recent developments in liquid biopsy and endoscopy approaches. Spectrochim. Acta A Mol. Biomol. Spectrosc..

[20] Balasundaram G., Krafft C., Zhang R., Dev K., Bi R., Moothanchery M., Popp J., Olivo M. (2021). Biophotonic technologies for assessment of breast tumor surgical margins—A review. J. Biophotonics.

[21] Haruki T., Yonezawa S., Koizumi K., Yoshida Y., Watanabe T.M., Fujita H., Oshima Y., Oku M., Taketani A., Yamazaki M. (2022). Application of the Dynamical Network Biomarker Theory to Raman Spectra. Biomolecules.

[22] Sakaue T., Hamaguchi M., Aono J., Nakashiro K.I., Shikata F., Kawakami N., Oshima Y., Kurata M., Nanba D., Masumoto J. (2020). Valve Interstitial Cell-Specific Cyclooxygenase-1 Associated with Calcification of Aortic Valves. Ann. Thorac. Surg..

[23] Akagi Y., Mori N., Kawamura T., Takayama Y., Kida Y.S. (2021). Non-invasive cell classification using the Paint Raman Express Spectroscopy System (PRESS). Sci. Rep..

[24] Asaoka R., Kiyomatsu H., Miura H., Jono A., Kinoshita T., Takao M., Katagiri T., Oshima Y. (2022). Prognostic potential and pathological validation of a diagnostic application using Raman spectroscopy in the characterization of degenerative changes in the cartilage of the humeral head. J. Biomed. Opt..

[25] Kneipp K., Kneipp H. (2006). Single Molecule Raman Scattering. Appl. Spectrosc..

[26] Puppels G.J., de Mul F.F., Otto C., Greve J., Robert-Nicoud M., Arndt-Jovin D.J., Jovin T.M. (1990). Studying single living cells and chromosomes by confocal Raman microspectroscopy. Nature.

[27] Puppels G.J., Garritsen H.S., Segers-Nolten G.M., de Mul F.F., Greve J. (1991). Raman microspectroscopic approach to the study of human granulocytes. Biophys. J..

[28] Baraga J.J., Feld M.S., Rava R.P. (1992). In situ optical histochemistry of human artery using near infrared Fourier transform Raman spectroscopy. Proc. Natl. Acad. Sci. USA.

[29] Shim M.G., Song L.M., Marcon N.E., Wilson B.C. (2000). In vivo near-infrared Raman spectroscopy: Demonstration of feasibility during clinical gastrointestinal endoscopy. Photochem. Photobiol..

[30] Molckovsky A., Song L.M., Shim M.G., Marcon N.E., Wilson B.C. (2003). Diagnostic potential of near-infrared Raman spectroscopy in the colon: Differentiating adenomatous from hyperplastic polyps. Gastrointest. Endosc..

[31] Haka A.S., Volynskaya Z., Gardecki J.A., Nazemi J., Lyons J., Hicks D., Fitzmaurice M., Dasari R.R., Crowe J.P., Feld M.S. (2006). In vivo margin assessment during partial mastectomy breast surgery using raman spectroscopy. Cancer Res..

[32] Oshima Y., Sato H., Zaghloul A., Foulks G.N., Yappert M.C., Borchman D. (2009). Characterization of human meibum lipid using raman spectroscopy. Curr. Eye Res..

[33] Hamada K., Fujita K., Smith N.I., Kobayashi M., Inouye Y., Kawata S. (2008). Raman microscopy for dynamic molecular imaging of living cells. J. Biomed. Opt..

[34] Saar B.G., Freudiger C.W., Reichman J., Stanley C.M., Holtom G.R., Xie X.S. (2010). Video-rate molecular imaging in vivo with stimulated Raman scattering. Science.

[35] Ozeki Y., Kitagawa Y., Sumimura K., Nishizawa N., Umemura W., Kajiyama S., Fukui K., Itoh K. (2010). Stimulated Raman scattering microscope with shot noise limited sensitivity using subharmonically synchronized laser pulses. Opt. Express.

[36] Satoh S., Otsuka Y., Ozeki Y., Itoh K., Hashiguchi A., Yamazaki K., Hashimoto H., Sakamoto M. (2014). Label-free visualization of acetaminophen-induced liver injury by high-speed stimulated Raman scattering spectral microscopy and multivariate image analysis. Pathol. Int..

[37] Egawa M., Tokunaga K., Hosoi J., Iwanaga S., Ozeki Y. (2016). In situ visualization of intracellular morphology of epidermal cells using stimulated Raman scattering microscopy. J. Biomed. Opt..

[38] Minamikawa T., Niioka H., Araki T., Hashimoto M. (2011). Real-time imaging of laser-induced membrane disruption of a living cell observed with multifocus coherent anti-Stokes Raman scattering microscopy. J. Biomed. Opt..

[39] Hashimoto T., Segawa H., Okuno M., Kano H., Hamaguchi H.O., Haraguchi T., Hiraoka Y., Hasui S., Yamaguchi T., Hirose F. (2012). Active involvement of micro-lipid droplets and lipid-droplet-associated proteins in hormone-stimulated lipolysis in adipocytes. J. Cell Sci..

[40] Huff T.B., Shi Y., Sun W., Wu W., Shi R., Cheng J.X. (2011). Real-time CARS imaging reveals a calpain-dependent pathway for paranodal myelin retraction during high-frequency stimulation. PLoS ONE.

[41] Imitola J., Côté D., Rasmussen S., Xie X.S., Liu Y., Chitnis T., Sidman R.L., Lin C.P., Khoury S.J. (2011). Multimodal coherent anti-Stokes Raman scattering microscopy reveals microglia-associated myelin and axonal dysfunction in multiple sclerosis-like lesions in mice. J. Biomed Opt..

[42] Shi L., Fung A.A., Zhou A. (2021). Advances in stimulated Raman scattering imaging for tissues and animals. Quant. Imaging Med. Surg..

[43] Suzuki Y., Kobayashi K., Wakisaka Y., Deng D., Tanaka S., Huang C.J., Lei C., Sun C.W., Liu H., Fujiwaki Y. (2019). Label-free chemical imaging flow cytometry by high-speed multicolor stimulated Raman scattering. Proc. Natl. Acad. Sci. USA.

[44] Nitta N., Iino T., Isozaki A., Yamagishi M., Kitahama Y., Sakuma S., Suzuki Y., Tezuka H., Oikawa M., Arai F. (2020). Raman image-activated cell sorting. Nat. Commun..

[45] Oshima Y., Shinzawa H., Takenaka T., Furihata C., Sato H. (2010). Discrimination analysis of human lung cancer cells associated with histological type and malignancy using Raman spectroscopy. J. Biomed. Opt..

[46] Bergholt M.S., Zheng W., Lin K., Ho K.Y., Teh M., Yeoh K.G., Yan So J.B., Huang Z. (2011). In vivo diagnosis of gastric cancer using Raman endoscopy and ant colony optimization techniques. Int. J. Cancer.

[47] Duraipandian S., Sylvest Bergholt M., Zheng W., Yu Ho K., Teh M., Guan Yeoh K., Bok Yan So J., Shabbir A., Huang Z. (2012). Real-time Raman spectroscopy for in vivo, online gastric cancer diagnosis during clinical endoscopic examination. J. Biomed. Opt..

[48] Lui H., Zhao J., McLean D., Zeng H. (2012). Real-time Raman spectroscopy for in vivo skin cancer diagnosis. Cancer Res..

[49] Ishigaki M., Maeda Y., Taketani A., Andriana B.B., Ishihara R., Wongravee K., Ozaki Y., Sato H. (2016). Diagnosis of early-stage esophageal cancer by Raman spectroscopy and chemometric techniques. Analyst.

[50] Féré M., Gobinet C., Liu L.H., Beljebbar A., Untereiner V., Gheldof D., Chollat M., Klossa J., Chatelain B., Piot O. (2020). Implementation of a classification strategy of Raman data collected in different clinical conditions: Application to the diagnosis of chronic lymphocytic leukemia. Anal. Bioanal Chem..

[51] Ma D., Shang L., Tang J., Bao Y., Fu J., Yin J. (2021). Classifying breast cancer tissue by Raman spectroscopy with one-dimensional convolutional neural network. Spectrochim. Acta A Mol. Biomol. Spectrosc..

[52] Li Z., Li Z., Chen Q., Ramos A., Zhang J., Boudreaux J.P., Thiagarajan R., Bren-Mattison Y., Dunham M.E., McWhorter A.J. (2021). Detection of pancreatic cancer by convolutional-neural-network-assisted spontaneous Raman spectroscopy with critical feature visualization. Neural Netw..

[53] Bouzy P., O’Grady S., Madupalli H., Tecklenburg M., Rogers K., Palombo F., Morgan M.P., Stone N. (2021). A time-course Raman spectroscopic analysis of spontaneous in vitro microcalcifications in a breast cancer cell line. Lab. Investig..

[54] Ogawa K., Oshima Y., Etoh T., Kaisyakuji Y., Tojigamori M., Ohno Y., Shiraishi N., Inomata M. (2021). Label-free detection of human enteric nerve system using Raman spectroscopy: A pilot study for diagnosis of Hirschsprung disease. J. Pediatr Surg..

[55] Aguiar R.P., Falcão E.T., Pasqualucci C.A., Silveira L. (2022). Use of Raman spectroscopy to evaluate the biochemical composition of normal and tumoral human brain tissues for diagnosis. Lasers Med. Sci..

[56] Liu Z., Zhang P., Wang H., Zheng B., Sun L., Zhang D., Fan J. (2022). Raman Spectrum-Based Diagnosis Strategy for Bladder Tumor. Urol. Int..

[57] Shaikh R., Daniel A., Lyng F.M. (2023). Raman Spectroscopy for Early Detection of Cervical Cancer, a Global Women’s Health Issue-A Review. Molecules.

[58] Nicolson F., Kircher M.F., Stone N., Matousek P. (2021). Spatially offset Raman spectroscopy for biomedical applications. Chem. Soc. Rev..

[59] Hanna K., Krzoska E., Shaaban A.M., Muirhead D., Abu-Eid R., Speirs V. (2022). Raman spectroscopy: Current applications in breast cancer diagnosis, challenges and future prospects. Br. J. Cancer.

[60] Zhang Y., Ren L., Wang Q., Wen Z., Liu C., Ding Y. (2022). Raman Spectroscopy: A Potential Diagnostic Tool for Oral Diseases. Front. Cell Infect. Microbiol..

[61] Liu K., Zhao Q., Li B., Zhao X. (2022). Raman Spectroscopy: A Novel Technology for Gastric Cancer Diagnosis. Front. Bioeng. Biotechnol..

[62] Huang L., Sun H., Sun L., Shi K., Chen Y., Ren X., Ge Y., Jiang D., Liu X., Knoll W. (2023). Rapid, label-free histopathological diagnosis of liver cancer based on Raman spectroscopy and deep learning. Nat. Commun..

[63] Li J., Li Y., Li P., Zhang Y., Du L., Wang Y., Zhang C., Wang C. (2022). Exosome detection via surface-enhanced Raman spectroscopy for cancer diagnosis. Acta Biomater..

[64] Liu H., Gao X., Xu C., Liu D. (2022). SERS Tags for Biomedical Detection and Bioimaging. Theranostics.

[65] Fornasaro S., Sergo V., Bonifacio A. (2022). The key role of ergothioneine in label-free surface-enhanced Raman scattering spectra of biofluids: A retrospective re-assessment of the literature. FEBS Lett..

[66] Dey P., Blakey I., Stone N. (2020). Diagnostic prospects and preclinical development of optical technologies using gold nanostructure contrast agents to boost endogenous tissue contrast. Chem. Sci..

[67] Song H., Dong C., Zhang X., Wu W., Chen C., Ma B., Chen F., Chen C., Lv X. (2022). Rapid identification of papillary thyroid carcinoma and papillary microcarcinoma based on serum Raman spectroscopy combined with machine learning models. Photodiagn. Photodyn. Ther..

[68] Lee S., Oh J., Lee K., Cho M., Paulson B., Kim J.K. (2022). Diagnosis of Ischemic Renal Failure Using Surface-Enhanced Raman Spectroscopy and a Machine Learning Algorithm. Anal. Chem..

[69] Qi Y., Zhang G., Yang L., Liu B., Zeng H., Xue Q., Liu D., Zheng Q., Liu Y. (2022). High-Precision Intelligent Cancer Diagnosis Method: 2D Raman Figures Combined with Deep Learning. Anal. Chem..

[70] Blake N., Gaifulina R., Griffin L.D., Bell I.M., Thomas G.M.H. (2022). Machine Learning of Raman Spectroscopy Data for Classifying Cancers: A Review of the Recent Literature. Diagnostics.

[71] Chen M., Feng X., Fox M.C., Reichenberg J.S., Lopes F.C.P.S., Sebastian K.R., Markey M.K., Tunnell J.W. (2022). Deep learning on reflectance confocal microscopy improves Raman spectral diagnosis of basal cell carcinoma. J. Biomed. Opt..

[72] Cheng X., Liang H., Li Q., Wang J., Liu J., Zhang Y., Ru Y., Zhou Y. (2022). Raman spectroscopy differ leukemic cells from their healthy counterparts and screen biomarkers in acute leukemia. Spectrochim. Acta A Mol. Biomol. Spectrosc..

[73] Cherkasova O.P., Nazarov M.M., Sapozhnikov D.A., Man’kova A.A., Fedulova E.V., Volodin V.A., Minaeva V.A., Minaev B.F., Baryshnikov G.V. (2010). Vibrational spectra of corticosteroid hormones in the terahertz range. Laser Applications in Life Sciences.

[74] Minaeva V.A., Minaev B.F., Baryshnikov G.V., Surovtsev N.V., Cherkasova O.P., Tkachenko L.I., Karaush N.N., Stromylo E.V. (2015). Temperature effects in low-frequency Raman spectra of corticosteroid hormones. Opt. Spectrosc..

[75] Minaeva V.A., Cherkasova O., Minaev B.F., Baryshnikov G.V., Khmara A.V. (2015). Features of terahertz adsorption and Raman scattering of mineralocorticoid hormones. Bull. Russ. Acad. Sci. Phys..

[76] Minaeva V.A., Minaev B.F., Hovorun D.M. (2008). Vibrational spectra of the steroid hormones, estradiol and estriol, calculated by density functional theory: The role of low-frequency vibrations. Ukr. Biokhim. Zh..

[77] Swain R.J., Jell G., Stevens M.M. (2008). Non-invasive analysis of cell cycle dynamics in single living cells with Raman micro-spectroscopy. J. Cell Biochem..

[78] Neugebauer U., Bocklitz T., Clement J.H., Krafft C., Popp J. (2010). Towards detection and identification of circulating tumor cells using Raman spectroscopy. Analyst.

[79] Kumamoto Y., Taguchi A., Smith N.I., Kawata S. (2012). Deep ultraviolet resonant Raman imaging of a cell. J. Biomed. Opt..

[80] Lin H.H., Li Y.C., Chang C.H., Liu C., Yu A.L., Chen C.H. (2012). Single nuclei Raman spectroscopy for drug evaluation. Anal. Chem..

[81] Movasaghi Z., Rehaman S., Rehman I.U. (2007). Raman Spectroscopy of biological Tissues. Appl. Spectrosc. Rev..

[82] Harvey T.J., Faria E.C., Henderson A., Gazi E., Ward A.D., Clarke N.W., Brown M.D., Snook R.D., Gardner P. (2008). Spectral discrimination of live prostate and bladder cancer cell lines using Raman optical tweezers. J. Biomed. Opt..

[83] Wood B.R., Hammer L., Davis L., McNaughton D. (2005). Raman microspectroscopy and imaging provides insights into heme aggregation and denaturation within human erythrocytes. J. Biomed. Opt..

[84] Ishigaki M., Kashiwagi S., Wakabayashi S., Hoshino Y. (2021). In situ assessment of mitochondrial respiratory activity and lipid metabolism of mouse oocytes using resonance Raman spectroscopy. Analyst.

[85] Okada M., Smith N.I., Palonpon A.F., Endo H., Kawata S., Sodeoka M., Fujita K. (2012). Label-free Raman observation of cytochrome c dynamics during apoptosis. Proc. Natl. Acad. Sci. USA.

[86] Abramczyk H., Brozek-Pluska B., Kopeć M. (2022). Double face of cytochrome c in cancers by Raman imaging. Sci. Rep..

[87] Haka A.S., Shafer-Peltier K.E., Fitzmaurice M., Crowe J., Dasari R.R., Feld M.S. (2005). Diagnosing breast cancer by using Raman spectroscopy. Proc. Natl. Acad. Sci. USA.

[88] You A.Y.F., Bergholt M.S., St-Pierre J.P., Kit-Anan W., Pence I.J., Chester A.H., Yacoub M.H., Bertazzo S., Stevens M.M. (2017). Raman spectroscopy imaging reveals interplay between atherosclerosis and medial calcification in the human aorta. Sci. Adv..

[89] Molino G., Dalpozzi A., Ciapetti G., Lorusso M., Novara C., Cavallo M., Baldini N., Giorgis F., Fiorilli S., Vitale-Brovarone C. (2019). Osteoporosis-related variations of trabecular bone properties of proximal human humeral heads at different scale lengths. J. Mech. Behav. Biomed. Mater..

[90] Falgayrac G., Farlay D., Ponçon C., Béhal H., Gardegaront M., Ammann P., Boivin G., Cortet B. (2021). Bone matrix quality in paired iliac bone biopsies from postmenopausal women treated for 12 months with strontium ranelate or alendronate. Bone.

[91] Ishimaru Y., Oshima Y., Imai Y., Iimura T., Takanezawa S., Hino K., Miura H. (2018). Raman Spectroscopic Analysis to Detect Reduced Bone Quality after Sciatic Neurectomy in Mice. Molecules.

[92] Kumar R., Grønhaug K.M., Afseth N.K., Isaksen V., de Lange Davies C., Drogset J.O., Lilledahl M.B. (2015). Optical investigation of osteoarthritic human cartilage (ICRS grade) by confocal Raman spectroscopy: A pilot study. Anal. Bioanal. Chem..

[93] Kroupa K.R., Wu M.I., Zhang J., Jensen M., Wong W., Engiles J.B., Schaer T.P., Grinstaff M.W., Snyder B.D., Bergholt M.S. (2022). Raman needle arthroscopy for in vivo molecular assessment of cartilage. J. Orthop. Res..

[94] Kar S., Jaswandkar S.V., Katti K.S., Kang J.W., So P.T.C., Paulmurugan R., Liepmann D., Venkatesan R., Katti D.R. (2022). Label-free discrimination of tumorigenesis stages using in vitro prostate cancer bone metastasis model by Raman imaging. Sci. Rep..

[95] Fousková M., Vališ J., Synytsya A., Habartová L., Petrtýl J., Petruželka L., Setnička V. (2023). In vivo Raman spectroscopy in the diagnostics of colon cancer. Analyst.

[96] Kang Z., Li Y., Liu J., Chen C., Wu W., Chen C., Lv X., Liang F. (2023). H-CNN combined with tissue Raman spectroscopy for cervical cancer detection. Spectrochim. Acta A Mol. Biomol. Spectrosc..

[97] David S., Tran T., Dallaire F., Sheehy G., Azzi F., Trudel D., Tremblay F., Omeroglu A., Leblond F., Meterissian S. (2023). In situ Raman spectroscopy and machine learning unveil biomolecular alterations in invasive breast cancer. J. Biomed. Opt..

[98] Lima A.M.F., Daniel C.R., Pacheco M.T.T., de Brito P.L., Silveira L. (2022). Discrimination of leukemias and non-leukemic cancers in blood serum samples of children and adolescents using a Raman spectral model. Lasers Med. Sci..

[99] Aihara K., Liu R., Koizumi K., Liu X., Chen L. (2022). Dynamical network biomarkers: Theory and applications. Gene.

[100] Chen L., Liu R., Liu Z.-P., Li M., Aihara K. (2021). Detecting early-warning signals for sudden deterioration of complex diseases by dynamical network biomarkers. Sci. Rep..

[101] Liu R., Wang X., Aihara K., Chen L. (2013). Early diagnosis of complex diseases by molecular biomarkers, network biomarkers, and dynamical network biomarkers. Med. Res. Rev..

[102] Koizumi K., Oku M., Hayashi S., Inujima A., Shibahara N., Chen L., Igarashi Y., Tobe K., Saito S., Kadowaki M. (2019). Identifying pre-disease signals before metabolic syndrome in mice by dynamical network biomarkers. Sci. Rep..

[103] Shi J., Aihara K., Chen L. (2021). Dynamics-based data science in biology. Natl. Sci. Rev..

